# Oral Microbiome Community Composition in Head and Neck Squamous Cell Carcinoma

**DOI:** 10.3390/cancers15092549

**Published:** 2023-04-29

**Authors:** William J. Benjamin, Kai Wang, Katherine Zarins, Emily Bellile, Freida Blostein, Ilona Argirion, Jeremy M. G. Taylor, Nisha J. D’Silva, Steven B. Chinn, Samara Rifkin, Maureen A. Sartor, Laura S. Rozek

**Affiliations:** 1 University of Michigan Medical School, Ann Arbor, MI 48109, USA; 2 Department of Computational Medicine and Bioinformatics, University of Michigan, Ann Arbor, MI 48109, USA; 3 Department of Environmental Health Sciences, University of Michigan, Ann Arbor, MI 48109, USA; 4 Department of Biostatistics, University of Michigan, Ann Arbor, MI 48109, USA; 5 Department of Epidemiology, University of Michigan, Ann Arbor, MI 48109, USA; 6 Division of Cancer Epidemiology and Genomics, National Cancer Institute, Bethesda, MA 20814, USA; 7 Department of Periodontics and Oral Medicine, University of Michigan, Ann Arbor, MI 48109, USA; 8 Department of Otolaryngology—Head and Neck Surgery, University of Michigan, Ann Arbor, MI 48109, USA; 9 Department of Gastroenterology, University of Michigan, Ann Arbor, MI 48109, USA; 10 Medical Center Department of Oncology, Georgetown University, Washington, DC 20007, USA

**Keywords:** oral microbiome, head and neck cancer, microbiome community type, head and neck squamous cell carcinoma

## Abstract

**Simple Summary:**

The impact of the oral microbiome on head and neck cancer is poorly understood. Better characterization of its impact may improve our understanding of the development of the disease and management of disease outcomes. This case–control study seeks to identify differences in the oral microbiome between patients who have head and neck cancer and controls who do not. Furthermore, we seek to identify types of microbial communities based upon abundance and compare those types with survival outcomes. We found that two commensal microbes that are associated with pathologic states when overgrown were more common in head and neck cancer cases than the controls. Furthermore, we identified two community types within our population. The community type with previously established pathogenic microbes had a lower yet non-significant hazard of death compared to the community with a higher abundance of commensal organisms.

**Abstract:**

The impact of the oral microbiome on head and neck cancer pathogenesis and outcomes requires further study. 16s rRNA was isolated and amplified from pre-treatment oral wash samples for 52 cases and 102 controls. The sequences were binned into operational taxonomic units (OTUs) at the genus level. Diversity metrics and significant associations between OTUs and case status were assessed. The samples were binned into community types using Dirichlet multinomial models, and survival outcomes were assessed by community type. Twelve OTUs from the phyla Firmicutes, Proteobacteria, and Acinetobacter were found to differ significantly between the cases and the controls. Beta-diversity was significantly higher between the cases than between the controls (*p* < 0.01). Two community types were identified based on the predominant sets of OTUs within our study population. The community type with a higher abundance of periodontitis-associated bacteria was more likely to be present in the cases (*p* < 0.01), in older patients (*p* < 0.01), and in smokers (*p* < 0.01). Significant differences between the cases and the controls in community type, beta-diversity, and OTUs indicate that the oral microbiome may play a role in HNSCC.

## 1. Introduction

More than 800,000 new cases of head and neck cancer (oral cavity, pharynx, and larynx) were diagnosed in 2020 globally, and there were over 400,000 deaths associated with the disease, most of which were head and neck squamous cell carcinoma (HNSCC) [1]. Due to the high global impact of HNSCC, there is a need for continued development of approaches for prevention and management of the disease to complement current efforts in alcohol and tobacco cessation and prevention of human papillomavirus (HPV) infection. The oral microbiome has emerged as a potential player in HNSCC; however, its role in the development, progression, and prognosis of the disease is not fully understood [2].

The oral microbiome consists of diverse microbial communities across microenvironments including tooth and epithelial surfaces. These microenvironments are diverse and distinct, each containing up to 40 species [3,4,5,6]. The overall function of the host microbiome is thought to contribute to homeostasis alongside contributing to tissue and immune system development [7]. Moreover, the host microbiome is thought to aid in immune function via colonization resistance, whereby commensal organisms outcompete potential pathogens within host microenvironments [8]. While the microbiome provides an important homeostatic mechanism for immune defense, oral microbiota dysbiosis has been implicated in multiple diseases including dental caries, periodontal disease, as well as oral, pharyngeal, and laryngeal cancers [5,9,10,11,12,13,14,15,16,17,18,19,20]. Notably, oral dysbiosis has been established as a risk factor independent of alcohol, tobacco, and HPV infection [21]. It is hypothesized that the relationship between HNSCC and the oral microbiome is due to the microbiota’s ability to alter inflammation within the microenvironment, alongside interfering with host cell signaling pathways involved in cellular proliferation, differentiation, and viability [14,22,23,24,25,26]. Okuyama et al., (2023) highlights the important synergistic contribution of the host environment, immune system, and oral microbiome that emphasizes a synergistic effect of *Porphyromonas gingivalis*, *Fusibacterium nucleatum*, and *Prevotella intermida* on the initiation of gingival squamous cell carcinoma through the induction of NF-κB-mediated immune responses that may promote cancer survival, oncogenic pathway activation, and aid in cancer cell migration and invasion [27].

Recent studies have helped establish microorganismal profiles of patients with HNSCC, made by culture-dependent or direct-sampling methods [10,11,12,19,20,28,29,30]. These studies have highlighted the role of microbial dysbiosis as an independent risk factor for HNSCC [21] alongside illustrating that increased abundance of commensal organisms *Corynebacterium* and *Kingella* is associated with a decreased risk of HNSCC [15]. Additionally, a microbiome–genome wide association study highlighted the dysbiosis relating to the abundance of Lachnoanaerobaculum along with two HACEK organisms, Aggregatibacter and Kingella, as being associated with genetic loci among oral squamous cell carcinoma patients [31]. Recent studies have also established a potential prognostic role for the microbiome in HNSCC, with a higher abundance of commensal organisms and a higher abundance of Fusobacterium being associated both at diagnosis and in the post-surgical period [32,33,34].

Our case–control study of oral rinse samples, which have been shown to be a reliable method of sampling the oral microbiome [35], seeks to add to the current body of literature through an assessment of diversity metrics and variations between HNSCC cases and controls. Furthermore, we aim to use community type analysis [36,37] to predict survival in HNSCC cases.

## 2. Materials and Methods

### 2.1. Study Population

#### 2.1.1. UM SPORE Project 3

Through the University of Michigan Head and Neck Specialized Program of Research Excellence (SPORE), newly diagnosed cases of head and neck cancer were recruited during 2008–2014. Eligible cases included individuals of at least 18 years of age, those who were not pregnant at the time of diagnosis, and those without a diagnosis of another primary cancer in the head and neck within the five years prior to enrollment. The subjects provided written informed consent, and the study was reviewed and approved by the University of Michigan institutional review board (HUM00065862).

#### 2.1.2. Case–Control Study

This study included 52 patients diagnosed with head and neck squamous cell carcinoma from a sample of 93 cases. The cases were recruited as a part of the prospective cohort study of the University of Michigan SPORE. The patients selected provided valid consent and were newly diagnosed, previously untreated, and older than 18 years of age. Forty-one cases were excluded from analysis due to having undergone chemoradiation prior to sample collection. One hundred and two age- and sex-matched controls were recruited through non-cancer otolaryngology clinics and provided valid consent, were English speakers, were older than 18 years of age, and had no previous history of non-melanoma cancer. The protocol was approved by the University of Michigan Medical School’s IRB (HUM00065862).

### 2.2. Measurements

#### 2.2.1. Lifestyle Information, Risk Factors, and Nutritional Information

The cases and controls were verbally administered lifestyle questionnaires that included questions about age, sex, height, weight, smoking history and alcohol use history. The cases completed a baseline nutrition survey upon entry into SPORE Project 3. The cases that did not complete a baseline nutritional survey were given one following their consent to the lifestyle questionnaire. The controls were given a nutritional survey following completion of the lifestyle questionnaire. The ACE-27 Comorbidity Index was calculated for the cases, and it provides a score between 0 and 3 based upon the severity of the comorbidity [38].

#### 2.2.2. Oral Wash Samples

Oral DNA samples were obtained using an Oragene-DNA collection kit (OG-500). The study participants were instructed to squirt 2.5 mL of 0.9% saline solution into their mouths and spit the sample into the provided kit following a few seconds of “swishing”. The DNA samples were stored at 4 C and prior to DNA extraction samples were incubated at 50 C in a bead bath for 2 h, per the manufacturer’s recommendations. The samples were randomized across the plates and lab personnel were blinded to the case–control status. The oral wash samples were used due to their availability, and we lacked direct tissue sampling.

#### 2.2.3. DNA Extraction and Quantification

DNA extraction was performed using a Eppedorf EpMotion liquid handling system and following this, the Qiagen MagAttract PowerMicrobiome kit (previously MoBio PowerMag Microbiome) protocol was used. After extraction, the samples (1 uL of template) were quantified using a Quant-iT PicoGreen dsDNA Assay kit (cat#: P7589).

#### 2.2.4. 16S rRNA Sequencing

The V4 region of the 16s rRNA gene was amplified from 372 samples using the dual indexing sequencing strategy developed by Dr. Patrick Schloss [39]. The samples included duplications plated to quality control and were randomized across plates. The PCR was performed, and the products were visualized using an E-Gel 96 with SYBR Safe DNA Gel Stain, 2% (Life technologies cat# G7208-02). The libraries were normalized using a SequalPrep Normalization Plate Kit (Life technologies cat # A10510-01) following the manufactur’s protocol for sequential elution. The concentration of the pooled samples was determined using a Kapa Biosystems Library Quantification kit for Illumina platforms (KapaBiosystems KK4824). The sizes of the amplicons in the library were determined using an Agilent Bioanalyzer High Sensitivity DNA analysis kit (cat# 5067-4626). The final library consisted of equal molar amounts from each of the plates, normalized to the pooled plate at the lowest concentration.

Library preparation for sequencing and the sequencing libraries were prepared according to Illumina’s protocol for Preparing Libraries for Sequencing on the MiSeq (part# 15039740 Rev. D) for 2 nM or 4 nM libraries. The positive and negative controls were sequenced and evaluated. If the library concentration was below 1 nM, an alternative method was used for denaturation [40]. PhiX and genomes were added into the 16s amplicon sequencing to create diversity. The sequencing reagents were prepared according to the Schloss SOP, custom read 1, and read 2, and index primers were added to the reagent cartridge. Sequencing was performed on the Illumina MiSeq platform, using MiSeq Reagent Kit V2 500 cycles (Illumina cat# MS102-2003), according to the manufacturer’s instructions with modifications found in the Schloss SOP. Accuprime High Fidelity Taq (Life Technologies cat # 12346094) was used instead of Accuprime Pfx supermix. FASTQ files were generated for paired end reads.

#### 2.2.5. Sequence Processing

Prior to processing, five samples were removed after they failed the quality control. A total of 367 bacterial genome sequences were processed using the mothur software package [41,42]. Sequence alignment was performed with the SILVA reference alignment (release 128). The sequences were binned into operational taxonomic units (OTUs) based on 97% similarity using the OptiClust method. To combine duplicate samples, the Pearson correlations between the OTU sequence counts were calculated. For duplicates with a correlation value greater than 0.9, average OTU counts were used in the analysis. For duplicates with a correlation value less than 0.9, the sample with the higher QC was used.

#### 2.2.6. Statistical Analysis

Demographic differences between the cases and controls were assessed using chi-square tests, Fisher’s exact tests, and t-tests. Bray–Curtis distances were calculated, and analysis of molecular variance was used to identify statistically significant differences between the microbiota of the cases and controls. Principal coordinates analysis and non-metric multidimensional scaling was used to visualize Bray–Curtis distances among the samples. EdgeR (version 3.30.3) was used with the quasi-likelihood method to assess statistically significant OTUs between the cases and controls, with a model controlling for age and sex and another model controlling for age, sex, and smoking. We considered a false discovery rate (FDR)-adjusted *p* value (q value) < 0.1 as significant. An HPV proxy variable was made by combining patients with positive HPV (i.e., HPV or p16 positivity regardless of site) and those missing HPV or p16 testing with an oropharyngeal site. Diversity metrics, including alpha diversity and beta diversity, were calculated using mothur. Alpha diversity was assessed for sex, smoking status, drinking status, tumor stage, tumor site, HPV proxy status, and treatment status. The Wilcoxon test was used to examine if beta diversity differed statistically between the cases and the controls. The samples were binned into two communities and labelled community 1 and community 2 based on the abundance of bacterial genera per sample using Dirichlet-multinomial models [43]. Demographic differences between community 1 and community 2 were assessed with chi-square tests, Fisher’s exact tests, and t-tests. To allow for the correction of potentially important variables in the survival analysis with minimal loss of degrees of freedom (allowing proper convergence of the model), direct surrogate variable analysis (dSVA) was used to discover hidden covariates within high dimensional data and produced two variables, V1 and V2 [44]. Multivariate analyses of 80-month overall survival were completed using a Cox-proportional hazards model adjusting for stage, HPV proxy, and V1, the main variable from the dSVA analysis. V2 was not included because it was found to correlate strongly with community type. Analyses were carried out using R Statistical Software (version 4.0.5, R Foundation).

## 3. Results

### 3.1. Study Population

Within the study population, the average age was 59.1, the average BMI was 29.7, and 17.9% of the population were female. No significant differences were found between cases and controls regarding age, BMI, sex, smoking history, or alcohol use history. Among the cases, 48.1% were stage 4, 17.3% were stage 3, and 34.6% were stage 1 or 2. Among the cases, 26.9% had laryngeal tumors, 34.6% had oral cavity tumors, 30.8% had oropharyngeal tumors, and 7.7% had hypopharyngeal, nasal or facial bone, nasopharyngeal, or unknown primary tumors. ACE-27 comorbidity status was measured for the cases, and 7.7% had a severe comorbidity score, 28.9% had a moderate comorbidity score, 17.3% had a mild comorbidity score, and 46.2% had a comorbidity score of zero. A total of 10.39% of cases were either HPV-positive or had an oropharyngeal disease site (Supplemental Table S1).

### 3.2. Associations among Operation Taxonomic Units (OTU) and HNSCC

In models adjusting for age, sex, and smoking, 6 OTUs were found to be significantly different between the cases and controls between the phyla firmicutes (*n* = 3), proteobacteria (*n* = 2), and Actinobacteria (*n* = 1). Among significant OTUs within the phylum firmicutes, a greater abundance of the family Lachnospiraceae that were unclassified at the genus level were found to be positively associated with HNSCC, while a greater abundance of the genera *Lactobacillus* and *Bacillus* were found to be negatively associated with HNSCC. Among the phylum Proteobacteria, a greater abundance of the genus *Eikenella* was found to be positively associated with HNSCC while a greater abundance of the genus *Acinetobacter* was found to be negatively associated with HNSCC. Among the phylum Actinobacteria, a greater abundance of the family Bifidobacteriaceae but not classified at the genus level was found to be negatively associated with HNSCC (Table 1).

### 3.3. Diversity Metrics

**Figure 1:**
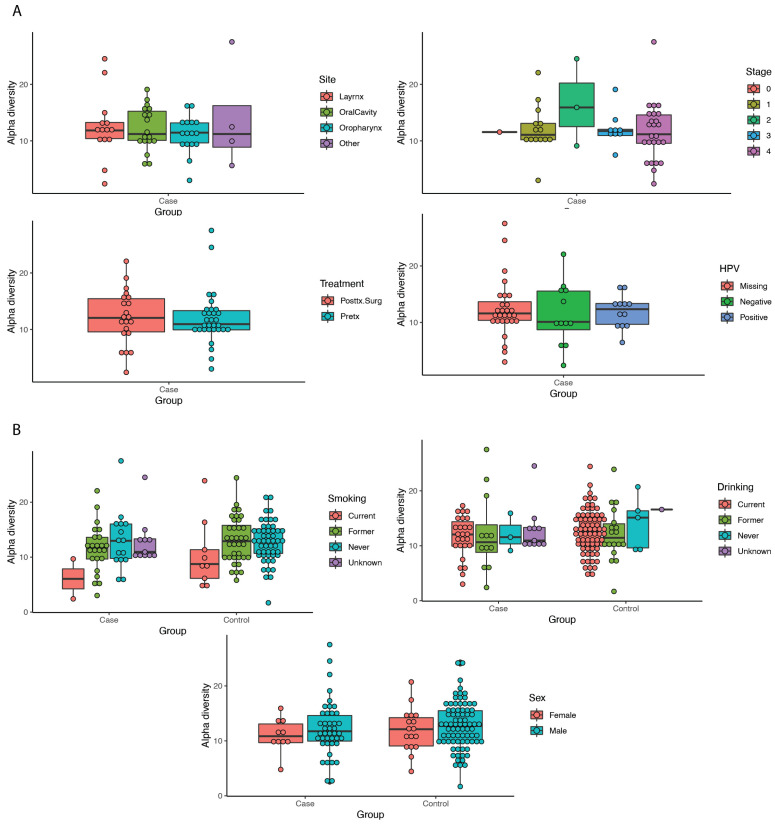
Alpha diversity measurements comparing case-only variables (**panel A**) and case and control variables (**panel B**).

Alpha diversity was calculated, and differences were tested for site, stage, HPV status, smoking, stage, and treatment, although no significant differences were found (all *p* > 0.05) (Figure 1). Beta diversity was calculated and the differences between pairs of controls and cases, cases and cases, and controls and controls were tested. Beta diversity was found to be higher among the cases compared to the controls (diff: 0.07, *p* < 0.0001) (Supplemental Figure S1 and Table 2).

### 3.4. Community and Survival Analyses

**Figure 2:**
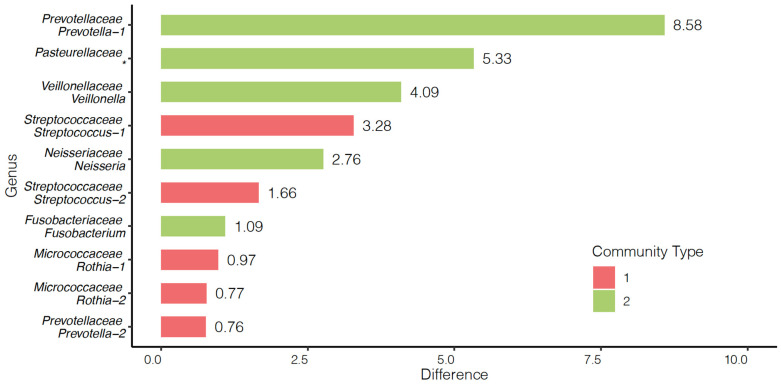
Community composition of community types 1 and 2. * OTU is measured to the Family level.

**Figure 3:**
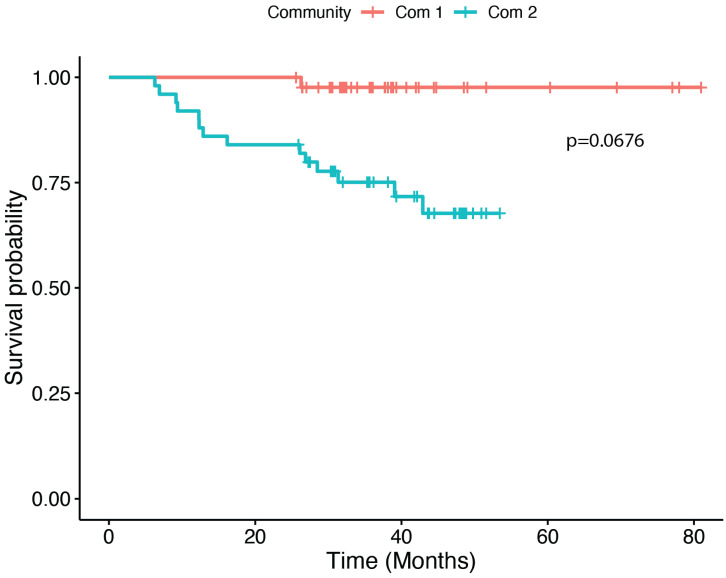
Kaplan–Meier curves comparing overall survival among the cases stratified by community type. *p*-value calculated from a Cox-proportional hazards model adjusting for stage, HPV, and V1, a surrogate variable from dSVA.

Two community types were identified as the predominant sets of OTUs within our study population. Community 1 was the predominant community for 29 cases and 80 controls, while community 2 was the predominant community for 23 cases and 22 controls. The predominant OTUs within community 1 were found to be *Streptococcus*, *Rothia*, and *Prevotella*. The predominant OTUs within community 2 were *Veilonella*, *Neisseria*, *Fusobacterium*, *Pasteurellaceae*, and a separate OTU from the genus Prevotella (Figure 2). The frequency of cases and controls within each community type differed significantly with 55.8% of cases and 80.2% of controls being sorted into community 1 (*p* = 0.0035). Community 1 and community 2 differed significantly by age (*p* = 0.0100) and smoking status (*p* = 0.0062) (Table 3). Within cases, a Cox proportional hazards model indicated that community 1 had a non-significantly longer survival compared to community 2 controlling for HPV proxy, V1 (from direct surrogate variable analysis (dSVA)), and stage (95.4% survival at 5 years for community 1 versus 55.5% for community 2) (*p* = 0.0676, Figure 3). Due to the difficult interpretation of the V1 variable, we also performed an alternative test without this covariate (i.e., with HPV proxy and stage) for comparison, which resulted in a similar finding for the difference in survival between communities (*p* = 0.0577).

## 4. Discussion

Our study found that the OTUs Lachnospiraceae and Eikenella were significantly more abundant in the HNSCC cases compared to the controls. Lachnospiraceae has been implicated in both the development and progression of periodontitis [45,46,47,48] and has been associated with germline genetic loci and oral dysbiosis in oral squamous cell carcinoma patients [31]. Furthermore, a significant difference in the abundance of Lachnospiraceae has been shown between oral non-betal quid using and non-smoking oral cancer cases and controls [49]. Eikenella, a HACEK organism, is a commensal organism in the oral cavity; however, overgrowth is associated with pathologic states including periodontitis and head and neck infections in patients with head and neck cancer [50,51,52]. Furthermore, it has previously been shown to be highly abundant in oral cavity cancer cases [53,54]. Aggreatibacter and Kingella, two other HACEK microbes, have also been found to be correlated with oral cavity cancer and periodontitis in additional studies [31,55,56,57]. Given the enrichment of Eikenella and Lachnospiraceae, it is biologically plausible that overgrowth may impact the development of HNSCC; however, given that we lack information on periodontal disease, these findings may be a result of confounding. Two previous case–control studies have implicated the role of the enrichment of an inflammatory microbiome consisting of periodontopathic bacteria including *Fusobacterium nucleatum*, *Prevotella tannerae*, *Prevotella intermedia*, and *Pseudomonas aeruginosa* in oral squamous cell carcinoma [58,59]. While our study did not illustrate enrichment of the same OTUs, it does further support the enrichment of inflammatory, periodontopathic organisms in HNSCC.

The beta diversity in our study was found to be higher among the cases compared to the controls, indicating a lesser degree of similarity among the cases than among the controls. This is consistent with other studies of the oral microbiome and indeed other molecular assays such as those for gene expression or epigenomics, which illustrated similar higher inter-individual differences for disease versus healthy populations [12,29,60]. Furthermore, alpha diversity was found to not differ significantly by site, stage, HPV status, smoking, alcohol use, treatment, or sex, which is consistent with other studies [15].

The existence and utility of enterotypes for classifying gut microbiomes suggests that stratification of the oral microbiome into community types my provide utility in our study [36,37]. Our community type analysis identified two predominant community groups in our entire sample which were found to significantly differ by age, smoking status, and case–control status with those in community 1, with individuals being younger, more likely to have never smoked, and more likely to be controls. This replicates the work of previous studies which have implicated tobacco use and age as factors that can impact the oral microbiota [15,33,61]. Additionally, our study found that our community types did not differ significantly by BMI, sex, site, stage, proxy HPV status, or alcohol use. Community 1 was more abundant in *Streptococcus* and *Rothia*, whereas community 2 had a higher abundance of *Fusobacterium* and *Prevotella*. *Fusobacterium nucleatum* (*F. nucleatum*) and *Prevotella intermedia* (*P. intermedia*) are periodontitis-associated species that have previously been implicated in oral squamous cell carcinoma carcinogenesis [27]. *F. nucleatum* may enhance the expression of cancer-related genes [62] and oncogenes including STAT3, JAK1, and MYC [63]. Furthermore, *F. nucleatum* has been show to increase the expression of markers of epithelial–mesenchymal transition and improve cancer cell invasiveness by enhancing matrix-metalloprotease and IL-8 expression in oral cancer cell lines [27,63]. *P. intermedia* secretes nucleases that can degrade neutrophil extracellular traps potentially enabling improved infiltration of periodontal tissues for *F. nucleatum* and *Porphyomonas gingivalis* [27,64].

We also sought to analyze whether community type was related to survival. Cases falling into community 1, which had larger abundances in the genera *Streptococcus* and *Rothia,* were found to have longer, albeit non-significant differences in overall survival when controlling for HPV proxy status, stage, and using surrogate variable analysis to control for other unknown sources of variability (*p* = 0.0676). While interesting, these results should be viewed with caution. Given sample size constraints, we were unable to appropriately stratify or control for multiple variables known to be associated with clinical survival outcomes, including site, age, comorbidities, and BMI. These results and their limitations highlight the importance of appropriately measuring and accounting for clinical and lifestyle predictors of survival in future investigations of the prognostic impact of the microbiome. While assumptions should be avoided regarding these survival results due to the aforementioned limitations, it is interesting that community 1, which had more abundant commensal organisms, had non-significantly higher survival than community 2 which had a higher abundance of *Prevotella* and *Fusobacterium*. These findings are in line with Chan et al. (2021) who found that an increased relative abundance of Rothia and Streptococcus 6 months post surgery for oral cavity carcinoma was associated with improved 3-year disease-specific survival [34]. Both *Rothia* and *Streptococcus* are known commensals in the oral microbiome and were found to be associated with healthy controls in a case–control study on chronic periodontitis [65]. Furthermore, *Streptococci* were the most abundantly represented genus in mucosal tissues in a study defining the healthy oral microbiome [65,66]. *Prevotella* and *Fusobacterium*, which were abundant in community type 2, were associated with chronic periodontitis in the same case–control study [65]. Furthermore, *Prevotella and Fusobacterium* have been implicated as having a synergistic effect with *Porphyromonas gingivalis* via NF-κB which may enable tumor survival and invasion in gingival squamous cell carcinoma [27]. However, Chan et al., (2022) did highlight that a higher abundance of *Fusobacterium* was associated with improved 3-year disease-specific survival in HNSCC and oral cavity squamous cell carcinoma in an unadjusted Kaplan–Meier analysis; however it is notable that Fusobacterium abundance was associated with lower T-stage and N-stage and less smoking, which may explain these results [33]. This finding was further supported by Neuzillet et al., (2021) who found that a higher abundance of *F. nucleatum* was associated with improved overall survival in oral cavity squamous cell carcinoma [32]. Chan and Neuzillet’s studies lend credence to the caution that should be paid towards our results, which may be confounded by clinical predictors of survival. Taken together, this highlights the importance of the study design for future prognostic studies of the microbiome and HNSCC. Our study was based on available data and samples; future studies should work toward a priori sampling and data acquisition that will allow for a stronger assessment of survival adjusting for appropriate confounding clinical, epidemiological, and demographical variables [67].

Our study is limited by multiple factors. First, our samples were not collected at the same time point for each patient, which is important in that the microbiome may change throughout the progression of disease. Additionally, we lack patient history of dental caries and periodontitis. Since many patients with HNSCC have comorbid dental diseases and our control group may have less periodontal disease, it may serve as an important unmeasured confounder in this study and limits our ability to derive conclusions. Additionally, we lack HPV testing on all our cases. We partially addressed this by building an HPV-proxy variable, which included all patients who were HPV-positive from all sites combined with oropharyngeal squamous cell carcinoma patients. However, given the prominent impact of HPV on HNSCC development, microbiota correlations, and clinical outcomes, further study with complete HPV data is needed [33]. Additionally, due to sample size constraints, we were unable to adjust for or stratify by known confounding variables in our survival analysis, including age, site, comorbidities, smoking, and BMI. This is particularly important for the disease subsite, as large variability exists across sites and the utilization of the salivary microbiome across all sites may be problematic. While some of this variation may have been addressed with our surrogate variable analysis, overall, our survival results should be viewed with caution. This study was completed on the available data and samples. Due to this, we only had oral rinse samples and lacked direct tissue sampling. Given established differences in microbial community structure across the sampling types, our study would have been improved through the presence of paired samples [68]. We are limited in generalizability as a single-center study, in particular given noted geographic variation in the oral microbiome [69]. Lastly, since this is not a primary study base, we cannot be certain that the controls are representative of the population that gave rise to the cases.

## 5. Conclusions

This case–control study of the oral microbiome in HNSCC found that the cases were associated with a higher abundance of the families Lachnospiraceae and Eikenella, which have previously been associated with periodontitis, thereby implicating a healthy oral microbiome in the prevention of HNSCC. Furthermore, beta diversity was found to be higher among the cases than among the controls. Lastly, our community type analysis identified two community types across the cases and controls. The community with a higher abundance of periodontitis-associated Fusobacterium and Prevotella was more likely to be older, smoke, and be a case compared to the community type with a higher abundance of commensal organisms including Streptococcus and Rothia. The community with a higher abundance of periodontitis-associated organisms appears to have near-significantly poorer overall survival; however this analysis may be confounded by site, age, and comorbidities. Additional analysis across different taxonomic groupings may help further elucidate the role of the oral microbiome in the risk and prognosis of HNSCC. Furthermore, future studies investigating the prognostic impact of the microbiome should be designed a priori in a manner that allows for controlling of clinical, epidemiological, and demographical factors that may impact survival.

## Figures and Tables

**Table 1:** Statistically significant operational taxonomic units between the cases and controls in the models controlling for age, sex, and smoking.

Bacterial Genus (Phylum/Class/Order/Family)	LogFc	*p*-Value	FDR
*Lactobacillus* (Firmicutes/Bacilli/Lactobacillales/Lactobacillaceae)	−7.72	0.0001	0.0082
*Unclassified* (Firmicutes/Clostridia/Clostridiales/Lachnospiraceae)	3.92	0.0020	0.0939
*Bacillus* (Firmicutes/Bacilli/Bacillales/Bacillaceae_1/)	−3.72	0.0022	0.0939
*Acinetobacter* (Proteobacter/Gammaproteobacteria/Pseudomonadales/Moraxeaceae)	−3.62	2.71 × 10^−13^	7.1 × 10^−11^
*Eikenella* (Proteobacteria/Betaproteobacteria/Neisseriales/Neisseriaceae)	5.81	1.9 × 10^−9^	2.5 × 10^−7^
*Unclassified* (Actinobacteria/Actinobacteria/Bifidobacteriales/Bifidobacteriaceae)	−4.02	1.5 × 10^−6^	0.0001

**Table 2:** Beta diversity between cases vs. cases, cases vs. controls, and controls vs. controls.

Group 1	Group 2	Group 1 Median	Group 2 Median	*p*-Value	P-adj (Holm)
Cases vs. Cases	Controls vs. Controls	0.57	0.45	9.2 × 10^−27^	2.6 × 10^−26^
Cases vs. Cases	Cases vs. Controls	0.57	0.49	2.0 × 10^−9^	4.0 × 10^−9^
Controls vs. Controls	Cases vs. Controls	0.45	0.49	1.3 × 10^−3^	1.3 × 10^−3^

**Table 3:** Epidemiological and clinicopathological characteristics of community 1 and community 2.

	Community 1	Community 2	
Characteristics	(*n* = 109)	(*n* = 45)	*p*-Value
Age (mean ± S.D)	57.57 ± 11.18	62.67 ± 10.68	0.0100
BMI no. (%) ^a^			0.7820
<20	1 (1.02)	0 (0.00)	
20–25	21 (21.43)	11 (28.21)	
25–30	38 (38.78)	13 (33.33)	
>30	38 (38.78)	15 (38.46)	
Sex no. (%) ^b^			0.5372
Female	18 (16.67)	9 (20.93)	
Male	90 (83.33)	34 (79.07)	
Alcohol Use no. (%) ^c^			0.0770
Never	5 (5.15)	3 (7.69)	
Former	17 (17.53)	13 (33.33)	
Current	75 (77.32)	23 (58.97)	
Smoking Status no. (%) ^d^			0.0062
Never	55 (56.12)	11 (28.21)	
Former	38 (38.78)	22 (56.41)	
Current	5 (5.10)	6 (15.38)	
Case status			0.0035
Case	29 (26.61)	23 (51.11)	
Control	80 (73.39)	22 (48.89)	
Site			0.2341
Larynx	8 (27.59)	6 (26.09)	
Oral Cavity	7 (24.14)	11 (47.83)	
Oropharynx	11 (37.93)	5 (21.74)	
Hypopharynx	0 (0.00)	1 (4.35)	
Nasal cavity, sinus, or skull	1 (3.45)	0 (0.00)	
Unknown primary	2 (6.90)	0 (0.00)	
HPV Proxy Variable	11 (10.09)	5 (11.11)	0.8505
Stage			0.0822
1 and 2	13 (44.83)	5 (21.74)	
3 and 4	16 (55.17)	18 (78.26)	

^a^ 17 missing BMIs: Fisher’s exact test used; ^b^ 3 missing sex; ^c^ 18 missing values for alcohol use; ^d^ 17 missing smoking statuses: Fisher’s exact test used.

## Data Availability

Data are available upon request from the corresponding author. They are not publicly available due to data access restrictions requiring institutional review board approval from the University of Michigan for protected health information.

## Supplementary Materials

The following supporting information can be downloaded at: https://www.mdpi.com/article/10.3390/cancers15092549/s1. Figure S1: Violin plots of beta diversity between cases vs. cases, controls vs. controls, and cases vs. controls; Table S1: Epidemiological and clinicopathological characteristics of 52 head and neck squamous cell carcinoma cases and 102 controls.
