# Supplementary material for: Oral Microbiome Community Composition in Head and Neck Squamous Cell Carcinoma
Source: Cancers (Basel). 2023 Apr 29;15(9):2549. doi: 10.3390/cancers15092549 (PMC10177240; doi:10.3390/cancers15092549)
Supplement: Supplementary file 1 [file cancers-15-02549-s001.zip › cancers-2244575-supplementary.pdf]

cancers

MDPI

Supplementary Material: Oral Microbiome Community Composition in Head and Neck Squamous Cell Carcinoma

Table S1. Epidemiologic and clinicopathologic characteristics of 52 cases and controls.

Characteristics	Case (n = 52)	Control (n = 102)	P-Value
Age (mean±S.D)	59.15±13.10	59.01±10.24	0.9450
BMI no. (%)			0.6502
< 20	0 (0.00)	1 (1.04)	
20-25	12 (29.27)	20 (20.83)	
25-30	16 (39.02)	35 (36.46)	
> 30	13 (31.71)	40 (41.67)	
Sex no. (%)			0.7537
Female	10 (19.23)	17 (17.17)	
Male	42 (80.77)	82 (82.83)	
Alcohol Use no. (%)			0.2849
Never	3 (7.32)	5 (5.26)	
Former	12 (29.27)	18 (18.95)	
Current	26 (63.41)	72 (75.79)	
Smoking Status no. (%)			0.0895
Never	15 (36.59)	51 (53.13)	
Former	24 (58.54)	36 (37.50)	
Current	2 (4.88)	9 (9.38)	
Stage no. (%)			
1 or 2	18 (34.62)	-	
3	9 (17.31)	-	
4	25 (48.08)	-	
Site no. (%)			
Larynx	14 (26.92)	-	
Oral Cavity	18 (34.62)	-	
Oropharynx	16 (30.77)	-	
Hypopharynx	1 (1.92)	-	
Nasal Cavity, Sinus, or Skull	1 (1.92)	-	
Unknown Primary	2 (3.85)	-	
HPV Proxy Variable	16 (10.39)	-	
ACE Comorbidity Score			
None	24 (46.15)	-	
Mild	9 (17.31)	-	
Moderate	15 (28.85)	-	
Severe	4 (7.69)	-	

[a]17 missing BMI, Fisher's Exact Test used
[b]3 missing sex
[c]18 missing values for alcohol use
[d]17 missing smoking status, Fisher's Exact Test used

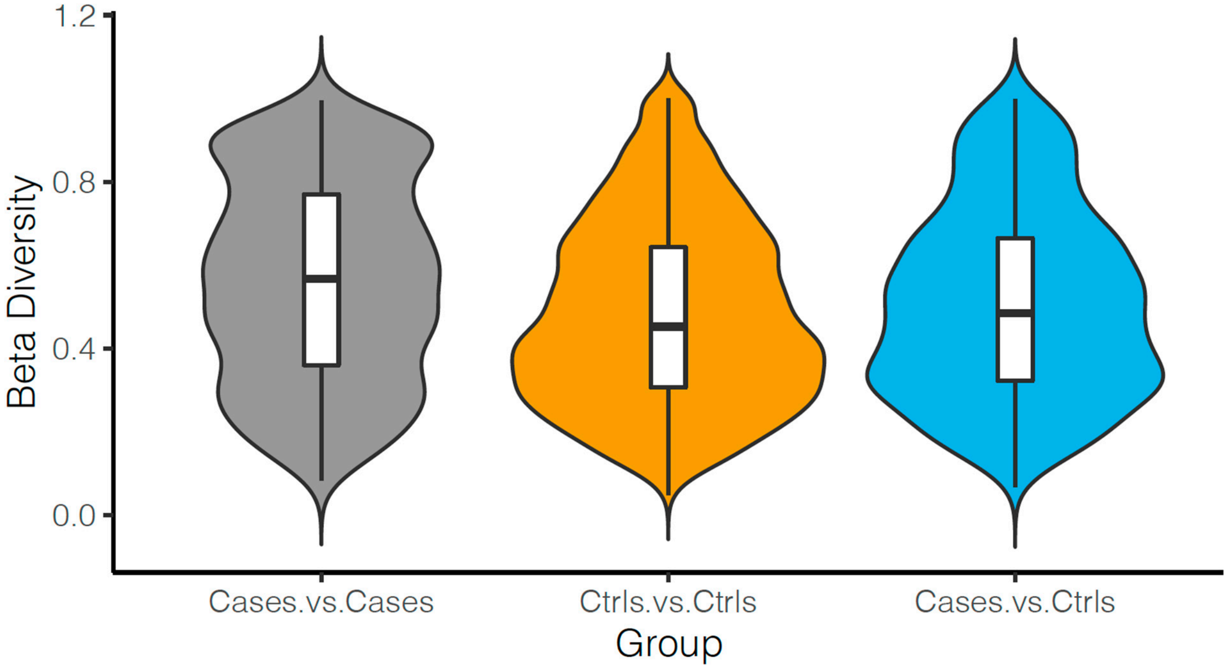

Figure S1. Violin Plots of Beta Diversity between cases vs. cases, controls vs. controls, and cases vs. controls.
